# Patients with Symptomatic Primary Hyperparathyroidism: An Anaesthetic Challenge

**Published:** 2009-08

**Authors:** Puneet Chopra, Sukanya Mitra

**Affiliations:** 1 Senior Resident, Department of Anaesthesia and Intensive Care, Government Medical College and Hospital, Chandigarh; 2 Professor, Department of Anaesthesia and Intensive Care, Government Medical College and Hospital, Chandigarh

**Keywords:** Hyperparathyroidism, Calcium, Anaesthesia

## Abstract

**Summary:**

Primary hyperparathyroidism is a disease characterized by hypercalcaemia attributable to autonomous overproduction of parathormone. Many patients with primary hyperparathyroidism are asymptomatic. Osteoporosis and nephrolithiasis are some of the major sequelae seen in the symptomatic patients. Parathyroidectomy is the only curative therapy. However anaesthetic management of such patients may be problematic with associated cardiac arrhythmias and skeletal muscle weakness. Low serum albumin and alteration in the acid base status in the perioperative period can affect the serum calcium level and thus adds to the existing problem. We present the successful anaesthetic management of a patient with primary hyperparathyroidism who initially presented with pathological fractures, and discuss the anaesthetic issues involved.

## Introduction

Calcium plays a central role in a large number of physiological actions that are essential for life. Of particular relevance to the anaesthetist are the effects of calcium on the myocardium, vascular smooth muscle and blood coagulation. The estimated incidence of hypercalcaemia is 1:1000 cases in males and 2-3:1000 cases in females, with primary hyperparathyroidism being the most common etiology. Primary hyperparathyroidism is the most common cause of hypercalcaemia in the outpatient setting, with many patients being asymptomatic. Left undiagnosed, it can lead to severe complications of hyperparathyroidism such as osteitis fibrosa cystica and nephrocalcinosis.^1^ We describe the successful anaesthetic management of a patient with primary hyperparathyroidism who initially presented with pathological fractures.

## Case Report

A 17-year-old female patient with a body weight of 40 kg presented with pain in left hip joint while walking for the last 6-8 months. There was no history of trauma. She was diagnosed with pathological fracture of left sided neck of femur with healed fracture right neck of femur, for which she was put on bilateral skin traction. The patient was investigated for pathological fractures which revealed following results: serum calcium 11.8 mg/dl, serum parathormone 302.90 pg/ml (normal value 15.0-68.30pg/ml), and alkaline phosphatase 2869 IU/l. Her serum electrolytes, blood urea, serum creatinine, albumin levels and electrocardiogram were within normal limits. A diagnosis of primary hyperparathyroidism was made on the basis of hypercalcaemia with increased parathormone levels. Ultrasound neck revealed presence of a hypo echoic mass of 3×1.1×1.2cm size just below the posteroinferioraspect of left lobe of thyroid. Technetium-99-m sestamibi parathyroid scan localized increased tracer uptake to posterior margin of left lobe of thyroid suggestive of left inferior parathyroid adenoma.

Once the diagnosis was confirmed, the patient was started on medical management with intravenous (IV) fluids and furosemide at dose of 40 mg IV 12 hourly. Subsequently her serum calcium came down to 8.2 mg/dl. Her hydration status and serum electrolytes were monitored during this forced saline diuresis therapy. The patient was then placed for left parathymidectomy. Preoperatively, the patient was kept nil per orally after 10 pm. Premeditation included ranitidine 150 mg, alprazolam 0.25mg orally night before and at 6a.m. on the morning of surgery. In the operating room, intravenous access was secured the patient was connected to multichannel monitor (S/5 Datex Ohmeda, Finland) and monitored for electrocardiogram (ECG), non-invasive blood pressure (NIBP), oxygen saturation, end tidal carbon dioxide (EtCO_2_) and neuromuscular function. The patient was pre-oxygenated for 3 minutes, and anaesthesia was induced with IV morphine 4.5 mg and IV thiopentone sodium 250 mg. Vecuronium bromide 5 mg IV was administered to facilitate tracheal intubation. Anaesthesia was maintained with 66% nitrous oxide in oxygen and isoflurane 0.5-1%. Patient's ECG was continuously monitored to detect any change in cardiac rhythm due to altered calcium metabolism. Further boluses of vecuronium bromide were given on the basis of neuromuscular monitoring. Patient's heart rate and blood pressure were stable throughout the period of surgery. End tidal CO_2_ was maintained between 32 and 36 mmHg. The surgery lasted for 90 minutes during which she received 1.5 L of crystalloids. After completion of surgery, the residual paralysis was reversed with neostigmine and glycopyrrolate in dosages of 2 mg and 0.4 mg respectively, with the guidance of neuromuscular monitor. During extubation the position of vocal cords was checked to assess any damage to recurrent laryngeal nerve. Intravenous calcium gluconate infusion was started slowly as a prophylactic measure and continued for 24 hours.

Postoperatively, the patient was kept in post anaesthesia care unit and was closely observed for signs and symptoms of hypocalcaemia. Serum calcium levels were checked regularly. On the second postoperative day, the patient was started on oral calcium. She received injectable paracetamol and tramadol for post-operative pain control. Her postoperative course was uneventful. For the pathological fracture, the patient was continued on skin traction and was discharged home with regular follow up at orthopaedics outpatient department.

## Discussion

Calcium is essential for many biological processes including cardiac automaticity, excitation contraction coupling, blood coagulation, neuronal conduction, synaptic transmission, hormone secretion and mitotic division.^1^ Extracellularcalcium occurs in three forms: as non-ionized protein bound (approximately 50%), as calcium-anion complexes (5%) and as ionized divalent cations (approximately 45%). It is the free (ionized) extracellular calcium concentration that mediates all the physiological effects, maintenance of which is affected by three main calciotropic hormones: parathyroid hormone (PTH), vitamin D and calcitonin.^2^ Most patients have single parathyroid adenoma (80%). Multiple gland hyperplasia is found in 10-20% of patients and parathyroid carcinoma is rare (1%).

Many patients with primary hyperparathyroidism are asymptomatic. In symptomatic patients common findings include renal calculi, bone pains, pathological fractures, skeletal muscle weakness or non-specific symptoms such as depression, lethargy, vague aches and pains. Cardiac manifestations include prolonged PR interval, short QT interval and systemic hypertension.^2^ The diagnosis of primary hyperparathyroidism is demonstrated by persistent hypercalcaemia in the presence of normal or elevated parathyroid hormone concentration.^3^,^4^

In our case, the patient presented with pathological fractures and bone pains. The patient was investigated for pathological fractures which subsequently demonstrated hypercalcaemia with primary hyperparathyroidism.

Preoperative localization of hypersecreting parathyroid gland has been attempted by many techniques. The most sensitive appears to be ultrasonography (which is operator dependant) and technetium-99m sestamibi tomographic nuclear scanning.^5^ In our case technetium-99 m sestamibi scan localized increased tracer activity in the left inferior parathyroid gland.

Primary hyperparathyroidism and the associated hypercalcaemia are treated initially by medical means followed by definitive surgical removal of the diseased or abnormal portions of parathyroid glands. Parathyroidectomy is the only curative treatment for primary hyperparathyroidism and is associated with 95% cure rate with minimal morbidity in the hands of an experienced endocrine surgeon.^6^ Intravenous fluids are the initial therapy for severe hypercalcaemia. Diuretic therapy should not be initiated until euvolemia is achieved. Loop diuretics depress the proximal tubular reabsorption of calcium and can increase the urinary calcium excretion by 200-250 mEq/day. Thiazide diuretics are avoided as these drugs may enhance renal tubular reabsorption of calcium. The risks of forced diuresis include cardiac decompensation, hypophosphataemia, hypokalaemia and hypomagnesaemia. Other treatment modalities include antiresorptive agents such as bisphosphonates, calcitonin and dialysis, which are reserved for the patients with renal failure.^3^,^7^ In our case, normocalcaemia was achieved with hydration and furosemide therapy.

Although there are no specific guidelines for the conduct of anaesthesia in patients with primary hyperparathyroidism, anaesthesia for hyperparathyroidism is not without problems. One needs to be vigilant about various factor that might alter serum calcium levels. It is important to correct malnutrition and low albumin levels in the preoperative period. Preexisting hypertension, which is more common in primary hyperparathyroidism, should be controlled if present. In the intraoperative period, special focus needs to be made on the acid base status and transfusion of large amounts of citrated blood, lest life threatening hypocalcaemia may ensue.^1^ Continuous ECG monitoring in these patients is imperative as hypercalcaemia may be associated with disturbance in cardiac rhythm, although there is evidence that QT interval may not be a reliable index of changes in serum calcium concentrations during anaesthesia.^8^ Coexisting skeletal muscle weakness may decrease the requirement of muscle relaxant in this group of patients. A reduction in the duration of action of rocuronium has been reported in a patient with normocalcaemic hyperparathyroidism, hence neuromuscular monitoring is mandatory, if available, in this group.^9^ Our patient was also monitored for neuromuscular blockade, although there was no alteration in the duration of action of muscle relaxant. Acidosis decreases calcium binding to albumin thus increasing the levels of ionized calcium, which can cause life threatening hypercalcaemia, hence it is important to maintain normocarbia. Our patient had severe osteoporosis and pathological fractures. This is a point of concern while managing patients with hypercalcaemia. Positioning in the operating table thus needs special care in these patients. One of the serious complications in these patients is recurrent laryngeal nerve injury. Hence assessment of vocal cord movement during extubation is imperative. Other severe problems encountered during surgery are bleeding and permanent hypoparathyroidism. Postoperative hypoparathyroidism needs to be monitored carefully. A high index of suspicion may avert life threatening respiratory failure and concomitant ECG changes. Serum calcium level usually normalizes by 3^rd^ – 4^th^ day and thus needs to be checked at regular postoperative intervals.^1^‐^3^ Patients with primary hyperparathyroidism usually develop less severe hypocalcaemia that is amenable to calcium therapy and it should be routinely initiated following subtotal parathyroidectomy.^10^ Our patient received IV calcium gluconate infusion forthe first 24 hours. Subsequently oral calcium therapy was instituted. Further, in the postoperative period, non-steroidal anti-inflammatory drugs should be avoided for pain control in case there is renal function impairment.^1^

In conclusion, it may be worth emphasizing that successful anaesthetic management of a patient with hyperparathyroidism requires vigilance for several factors that might potentiate adverse effects of hypo- and hypercalcaemia. These factors and their anaesthetic implications are summarized in Table 1. Adequate pre-operative assessment and preparation, close monitoring of the signs and symptoms of hypo- and hypercal-caemia, restoration and keeping ionized calcium within normal limits during perioperative period can go a long way in the successful anaesthetic management of patients with abnormal calcium metabolism.

**Table 1:** Anaesthetic considerations in surgery for primary hyperparathyroidism.

Phase of operation	Anatomic/physiologic issue	Anaesthetic implication
Preoperative	· Factors influencing serum calcium:	Correct nutrition and albumin level
	Malnutrition	
	Low albumin	
	· Hypertension	Control blood pressure
	· Renal function impairment	Assess renal function
Intraoperative	Altered acid-base status	Citrated blood transfusion
	(may lead to hypo- or hypercalcaemia)	Continuous ECG monitoring
	Skeletal muscle weakness	May need less muscle relaxant
		Neuromuscular monitoring
	Osteoporosis, pathological fractures	Care during positioning of patient
	Recurrent laryngeal nerve injury	Assess vocal cord movement during extubation
Postoperative	Postoperative hypoparathyroidism	Check serum calcium regularly till 3-4 postoperative days
		Calcium therapy(IV/oral) if needed
	Renal function impairment	Avoid NSAIDs
